# Anabolic steroids and the evaluation of patients with acute PM tendon rupture using microscopy and MRI

**DOI:** 10.1093/jscr/rjae126

**Published:** 2024-03-21

**Authors:** Alberto de Castro Pochini, Benno Ejnisman, Carlos V Andreoli, Paulo H S Lara, Ivan R B Godoy, Leandro M Ribeiro, Maria T Seixas, Paulo S Belangero, Debora C Hipolide

**Affiliations:** Department of Orthopaedic, Federal University of Sao Paulo, São Paulo 04022-000, Brazil; Department of Orthopaedic, Federal University of Sao Paulo, São Paulo 04022-000, Brazil; Department of Orthopaedic, Federal University of Sao Paulo, São Paulo 04022-000, Brazil; Department of Orthopaedic, Federal University of Sao Paulo, São Paulo 04022-000, Brazil; Department of Orthopaedic, Federal University of Sao Paulo, São Paulo 04022-000, Brazil; Department of Orthopaedic, Federal University of Sao Paulo, São Paulo 04022-000, Brazil; Department of Pathology, Federal University of Sao Paulo, São Paulo 04023-062, Brazil; Department of Orthopaedic, Federal University of Sao Paulo, São Paulo 04022-000, Brazil; Department of Psychobiology, Federal University of Sao Paulo, São Paulo 04724-000, Brazil

**Keywords:** tendinopathy, pectoralis major rupture, tendon rupture

## Abstract

This study presented a pioneering investigation of the changes in the magnetic resonance imaging images of pectoralis major muscle (PMM) tendon rupture. In all, 26 men were evaluated with acute total PMM rupture (<3 months since injury) with a mean age of 37.3 years (SD = 9.7 years) and 10 control patients with a mean age of 32.6 years (SD = 4.2 years). The evaluation of the tendon PMM injuries was based on the magnetic resonance imaging exam and the histological analysis. The magnetic resonance imaging of the surgically showed two (7.1%) contralateral sides were normal, 16 (57.1%) showed superior tendinopathy, and 10 (35.7%) had total tendinopathy. Inferior tendinopathy was not observed. The tendon histology revealed degenerative changes in 16 (66.7%) fragments, with 12 (50.0%) considered as mild (<25%), and four considered as (16.7%) high (>50.0%) tendinopathy. Total acute rupture of the PMM tendon among weightlifters might be associated with tendinous degeneration prior to injury.

## Introduction

The incidence of pectoralis major muscle (PMM) injury has increased considerably among weightlifters over the last 20 years. Failure to promptly recognize the injury described as “total PMM tendon rupture” among exercise practitioners typically leads to chronic injury phases with an important sequela (i.e. chest deformity) and decreased adduction strength, which usually bothers patients practicing weightlifting or other physical activities [1–24]. Treatment during the acute phase is typically and technically easier, although the literature describes positive results among patients operated on during the acute or chronic PMM injury stages [1–23]. In general, the treatment of acute cases involves the surgical fixation of the PMM tendon to the humerus with suture anchors, cortical buttons, or bone tunnels [1–3, 7, 9, 12–28]. In chronic cases (i.e. time since injury > 3 weeks) [25], the muscle can have difficulty being positioned in the region lateral to the biceps brachii, especially among weightlifters using anabolic steroids who present with complex injuries and medial retraction. In these cases, the use of autografts [6, 8, 9, 19–26] or allografts [15] is necessary, and the PMM tendon is reconstructed, which is technically more difficult but has positive results. We have observed that these patients have satisfactory esthetic and functional clinical results months after PMM reconstruction. Some studies have shown the use of magnetic resonance for injuries to the tendon of the PMM, but none have addressed possible previous changes to rupture [15–17, 21]. Until now we have not identified additional magnetic resonance imaging (MRI) signs suggesting evidence of tendon injury (tendinopathy), beyond those indicated on ultrasound [21]. In other tendons (e.g. the rotator cuff and calcaneal tendon) [24, 26], the MRI visualization of degenerative changes such as thickening and hyper signal within the tendon showing the proliferation of vessels and cells is possible. This study conducted a pioneering investigation of the changes in the MRI images of PMM tendon rupture compared with the contralateral side and among weightlifters and histologically analyzed ruptured PMM tendons to determine the presence of changes.

## Materials and methods

Between 2014 and 2018, 36 men patients were prospectively assessed, 26 with acute total PMM rupture (<3 months since injury) with a mean age of 37.3 years (SD = 9.7 years) and 10 control patients with a mean age of 32.6 years (SD = 4.2 years). Control patients were weightlifters without the use of anabolic steroids and without injuries related to weightlifting. All cases were caused by the bench press exercise and treated at. This study was approved by and registered at our institution under Ethical Committee CAAE number. The patients were evaluated using a Siemens 1.5 Tesla MRI system. Patients with PMM rupture were operated on using a previously described technique and a ruptured tendon sample was obtained from the injured side for histological evaluation. On the contralateral side to the PMM injury, chest MRI images were obtained to evaluate possible PMM tendinopathy. All patients were questioned regarding the association of rupture and the use of anabolic steroids.

### MRI evaluation

Patients and controls underwent a bilateral MRI protocol to directly evaluate the PMM tendon. All exams were performed using the same 1.5-T instrument (Siemens, Erlangen, Germany) with the parameters described in Table 1.

**Table 1:** MRI parameters.

**Pulse sequence**	**TR (ms)**	**TE (ms)**	**NEX**	**Matrix**	**Thickness (mm)**	**FOV (cm)**	**Bandwidth (Hz)**	**Echo train**
Coronal FSE T2FS	3000	49	1	512 × 256	4	22	250	6
Axial FSE T1	600	11	1	512 × 256	4	26	296	3
Axial FSE T2FS	3000	49	1	512 × 256	4	26	250	6
Sagittal FSE T2FS	3600	52	1	512 × 256	4	22	250	6
Coronal T1	500	15	1	512 × 256	4	30	122	1

TR, repetition time; TE, echo time; NEX, number of excitations; FOV, field-of-view; FSE, fast spin echo.

MRI scans were evaluated using Osirix v6.0 (Pixmeo, Bernex, Switzerland). A radiologist specialized in musculoskeletal imaging evaluated the images, considering a PMM injury to be present when abnormal morphology and hyper signal intensity were observed in the muscle and tendon structures on the T1-weighted sequences without fat saturation and the T2-weighted images with fat saturation (T2FS). Changes related to the tendinopathy of the PMM included the presence of tendon thickening and signal changes in at least two consecutive axial images on the T1 or T2FS sequences. Because of the difficulty of differentiating the sternal and clavicular components of the PMM tendon near its insertion, tendinopathy was also classified based on the location of the tendon injury: the upper half of the tendon, the lower half of the tendon, or the full extension of the PMM tendon.

### Optical microscopy

The sample consisted of 26 fragments from the patients undergoing surgery. In this study, only the tendons were analyzed via histological analysis of the remaining tendon attached to the retracted muscle (Table 2). The specimens were fixed in buffered formalin and embedded in paraffin for a hematoxylin and eosin analysis. The slides were evaluated by the General, Systemic, Forensic, and Bioethics Pathological Anatomy Department of our department.

**Table 2:** Classification used for histological analysis of tendons.

Tendinopathy	Classification
1 = degenerative changes (myxoid changes of the filamentous substance)	0 = absent
	1 = mild <25%
	2 = moderate 26%–50%
	3 = high >50%
2 = calcifications	0 = absent
	1 = present
3 = neovascularization	0 = absent
	1 = present
4 = fissures	0 = absent
	1 = present
	

### Statistical analysis

The numerical variables were described by mean and standard deviation and the categorical variables by absolute and relative frequencies. Generalized estimation equation models were adjusted considering the dependence between the sides of the same individual. The models were fitted with gamma distribution and log link function and the results presented by estimated mean values and 95% confidence intervals and the *P* values were obtained by multiple comparisons corrected by the Bonferroni method. The analyses were performed using SPSS® software version 19, adopting a significance level of 5%.

## Results

The control group of patients who underwent MRI consisted of 10 healthy participants with a mean age of 32.6 years (SD = 4.2 years). The group with previous PMM tendon injury was composed of 26 individuals with a mean age of 37.3 years (SD = 9.7 years). The bilateral measurements of all participants in the control group were obtained (10 measurements). The measurements of the case group were performed on the contralateral side (26 measurements) to determine the presence of degenerative changes because of long-term weightlifting. All 26 patients practiced weightlifting, specifically suffering a PMM injury during the bench press exercise. All cases had a history of anabolic steroid use, which is typical of PMM rupture cases engaged in recreational or competitive bench press exercise. All cases were men. Of the surgically treated patients in the case group, regarding the images, two (7.1%) contralateral sides were normal, 16 (57.1%) showed superior tendinopathy, and 10 (35.7%) had total tendinopathy. Inferior tendinopathy was not observed. The tendinopathy classification revealed that all control patients were classified as normal (Tables 3 and 4). Regarding the use of anabolic steroids, all use was reported by patients as use during the injury or prior to and close to the injury in a period of decreased drug effect around 1 month of withdrawal. The most used steroids were deca-durabolin (45%) and durateston (32%), stanozolol (17%), 23% others (primobolan, oxandrolone, boldenone, deposteron, nebido, trenbolone), and for some athletes, a mixture of both.

**Table 3:** PMM tendon measurements obtained via MRI in both cases and controls.

	Group			*P*-value		
	Control (G1)	Case: operated side (G2)	Case: contralateral side (G3)	G1 × G2	G1 × G3	G2 × G3
Tendon size	3.39 (2.90; 3.88)	4.39 (3.34; 5.44)	3.83 (3.52; 4.14)		0.273	
Larger PMM area	52.1 (38.7; 65.6)	51.2 (43.6; 58.8)	56.4 (50.1; 62.7)	>0.999		0.251
PMM volume	647.7 (447.3; 848.2)	615.7 (499.4; 731.9)	624.5 (543.6; 705.3)	>0.999		>0.999
Humeral area at PMM insertion	3.88 (3.39; 4.37)	4.46 (4.18; 4.73)	4.33 (4.08; 4.57)	0.134		0.136

**Table 4:** Tendinopathy findings from the pathological analysis of the tendon fragments of weightlifters with PMM tendon rupture.

**Tendinopathy**	
Degenerative changes (myxoid changes of the filamentous substance)	
Absent	8 (33.3%)
Mild (<25%)	12 (50.0%)
High (>50%)	4 (16.7%)
**Calcifications**	
Absent	24 (100.0%)
**Neovascularization**	
Absent	8 (33.3%)
Present	16 (66.7%)
**Fissures**	
Absent	22 (91.7%)
Present	2 (8.3%)
**Other findings**	
Foci of chronic inflammatory infiltrate in adjacent structures + foci of recent hemorrhage and lymphomononuclear infiltrate	2 (8.3%)
Recent hemorrhagic foci	2 (8.3%)
Inflammatory infiltrate	2 (8.3%)
Lymphomononuclear infiltrate	2 (8.3%)
Lymphomononuclear infiltrate + recent hemorrhages	2 (8.3%)
Lymphomononuclear infiltrate + tissue granulation	2 (8.3%)
Chronic lymphomononuclear infiltrate	2 (8.3%)

### Tendon samples

Two fragments could not be analyzed for insufficient tissue. The tendinopathy evaluations of the tendon fragments (Table 4) revealed degenerative changes in 16 (66.7%) fragments, with 12 (50.0%) considered as mild (<25%), and four considered as (16.7%) high (>50.0%) tendinopathy. None of the fragments showed calcifications, whereas 16 (66.7%) presented with neovascularization, and only two (8.3%) presented with a fissure. Other findings were observed, including four fragments (16.7%) with inflammatory infiltrate, 10 (41.7%) with lymphomononuclear infiltrate, three (25.0%) with the foci of recent hemorrhage, and two (8.3%) with the presence of granulation tissue.

## Discussion

The tendinopathy evaluations of the tendon fragments (Table 4) revealed degenerative changes in 16 (66.7%) fragments, with 12 (50.0%) considered as mild (<25%), and four considered as (16.7%) high (>50.0%) tendinopathy. As for the magnetic resonance study of the contralateral tendon in patients who ruptured the tendon of the PMM, the image can be used to better visualize changes such as thickening of the tendon in their most cranial or upper portions, but the better understanding of these changes takes time and careful observation of the image examination.

It is suspected that patients who use anabolic steroids show an important gain in postoperative muscle strength with a rapid improvement of postoperative discomfort. The accelerated improvement results from their rapid strength gain in, among other muscles, the deltoid muscle, the major agonist of shoulder elevation and movement. Thus, psychological factors are likely driving the search for muscle growth primarily during the postoperative period when intense muscle atrophy occurs, which represents improvements in the physical and psychological conditions of the current study’s weightlifters associated with the resumption of the use of anabolic steroids, typically between the second and third postoperative months. Thus, after 30 years of research on this type of injury, the use of prohibited substances (anabolic steroids) is ubiquitous because they are strongly associated with injuries when the load and overload of the PMM tendon increase as well as faster returns to improved muscle strength after shoulder injury compared with conventional patients. At the time of surgery, we were occasionally surprised by the degree of retraction of the PMM injury in patients using anabolic steroids, even when operated on within the first week of injury. In at least four cases, PMM tendon reconstruction using autografts was necessary to surgically treat acute injury. Thus, the surgeon should be prepared for cases in which the MRI suggests retractions that are more medial than the axillary line in bench press athletes and anabolic steroid users. In these cases, the patient and family should be alerted to the possible need for PMM reconstruction rather than repair. Why does PMM tendon injury not occur in weightlifting women as in men? [30]. Some believe that women do not use as many anabolic steroids or lift weights as heavy as men. For 30 years, we have been following the Brazilian Confederation of Basic Weightlifting, and several women compete with heavy weights relative to their body weights, similarly to men, as represented by lifting attempts with maximum load or bench press repetition. During clinical assessment, several women also reported the use of anabolic steroids similar to men who had ruptured tendons. Only the numerical value of the maximum tensile strength varies. In men, PMM injury loads ranging from 120 to 300 kg are reported after bench press attempts. These loads are uncommon among women; however, several female bench press athletes lift more than 120 kg in the higher body weight categories. Thus, a genetic or hormonal factor might confer women with a musculotendinous advantage to their upper limbs under maximum load compared with men. Another interesting factor is that reinjury because of overload is extremely uncommon among patients who undergo surgery. The majority of weightlifters change the intensity of their training and regular practice using maximum loads after surgery as well as become more aware of the effect of this overload on their bodies compared with before surgery. Although they resume competitive weightlifting, patients often report greater concern with and knowledge of the limitations of weightlifting overload. The clinical and imaging classification of the PMM injuries was based on the MRI exam, the histological analysis performed in this study, and a clinical follow-up study of total of 160 patients with PMM rupture that began in 2000, 25 of whom 95 underwent surgery (55 repairs and 40 PMM tendon reconstructions). Between 10% and 15% of our patients of the following cases 160 PMM injuries (acute and cronic) had histories of other musculo tendinous ruptures, including to the proximal biceps, distal biceps, triceps, or quadriceps. Some recreational practitioners have body-image disorders associated with anabolic steroid abuse related to childhood issues such as bullying and domestic violence according to psychologists from our sports medicine outpatient clinic [29, 30, 31, 32].

Another very serious complication that is less talked about as an adverse effect of the use of injectable anabolic steroids is pyomyositis, which every year kills some bodybuilding athletes [31].

## Conclusion

Total acute rupture of the PMM tendon among weightlifters might be associated with changes in the MRI signal and tendinous degeneration prior to injury, especially following bench press exercise, which is historically associated with the use of anabolic steroids.

## References

[1] Aarimaa V , Rantanen J, Heikkila J, Helttula I, Orava S. Rupture of the pectoralis major muscle. Am J Sports Med 2004;32:1256–62. 15262651 10.1177/0363546503261137

[2] Bak K , Cameron EA, Henderson IJ. Rupture of the pectoralis major: a meta-analysis of 112 cases. Knee Surg Sports Traumatol Arthrosc 2000;8:113–9. 10795675 10.1007/s001670050197

[3] Connell DA , Potter HG, Sherman MF, Wickiewicz TL. Injuries of the pectoralis major muscle: evaluation with MR imaging. Radiology 1999;210:785–91. 10207482 10.1148/radiology.210.3.r99fe43785

[4] de Castro PA , Andreoli CV, Belangero PS. Clinical considerations for the surgical treatment of pectoralis major muscle ruptures based on 60 cases: a prospective study and literature review. Am J Sports Med 2014;42:95–102. 24192390 10.1177/0363546513506556

[5] de Castro PA , Ejnisman B, Andreoli CV. Exact moment of tendon of pectoralis major muscle rupture captured on video. Br J Sports Med 2007;41:618–9. 17337486 10.1136/bjsm.2006.033563 PMC2465400

[6] de Castro PA , Ejnisman B, Andreoli CV. Pectoralis major muscle rupture in athletes: a prospective study. Am J Sports Med 2010;38:92–8. 19880715 10.1177/0363546509347995

[7] Egan TM , Hall H. Avulsion of the pectoralis major tendon in a weight lifter: repair using a barbed staple. Can J Surg 1987;30:434–5. 3664412

[8] Ejnisman B , Andreoli CV, Pochini AC, Carrera EF, Abdalla RJ, Cohen M. Ruptura do musculo peitoral maior em atletas. Rev Bras Ortop 2002;37:482–8.

[9] ElMaraghy AW , Devereaux MW. A systematic review and comprehensive classification of pectoralis major tears. J Shoulder Elbow Surg 2012;21:412–2. 21831661 10.1016/j.jse.2011.04.035

[10] Figueiredo EA , Terra BB, Cohen C. Footprint do tendão do peitoral maior: estudo anatômico. Rev Bras Ortop 2013;48:519–23. 31304163 10.1016/j.rboe.2013.12.009 PMC6565948

[11] Fleury AM , Silva AC, de Castro PA, Ejnisman B, Lira CA, Andrade MS. Isokinetic muscle assessment after treatment of pectoralis major muscle rupture using surgical or non-surgical procedures. Clinics (Sao Paulo) 2011;66:313–20. 21484052 10.1590/S1807-59322011000200022 PMC3059863

[12] Hanna CM , Glenny AB, Stanley SN, Caughey MA. Pectoralis major tears: comparison of surgical and conservative treatment. Br J Sports Med 2001;35:202–6. 11375884 10.1136/bjsm.35.3.202 PMC1724342

[13] Hart ND , Lindsey DP, McAdams TR. Pectoralis major tendon rupture: a biomechanical analysis of repair techniques. J Orthop Res 2011;29:1783–7. 21538507 10.1002/jor.21438

[14] Joseph TA , Defranco MJ, Weiker GG. Delayed repair of a pectoralis major tendon rupture with allograft: a case report. J Shoulder Elbow Surg 2003;12:101–4. 12610495 10.1067/mse.2003.128200

[15] Lee J , Brookenthal KR, Ramsey ML, Kneeland JB, Herzog R. MR imaging assessment of the pectoralis major myotendinous unit: an MR imaging- anatomic correlative study with surgical correlation. AJR Am J Roentgenol 2000;174:1371–5. 10789797 10.2214/ajr.174.5.1741371

[16] Miller MD , Johnson DL, Fu FH, Thaete FL, Blanc RO. Rupture of the pectoralis major muscle in a collegiate football player. Use of magnetic resonance imaging in early diagnosis. Am J Sports Med 1993;21:475–7. 8346766 10.1177/036354659302100325

[17] Ohashi K , El-Khoury GY, Albright JP, Tearse DS. MRI of complete rupture of the pectoralis major muscle. Skeletal Radiol 1996;25:625–8. 8915045 10.1007/s002560050148

[18] Pochini A , Ejnisman B, Andreoli CM, Cohen M. Reconstruction of the pectoralis major tendon using autologous grafting and cortical button attachment: description of the technique. Tech Shoulder Elbow Surg 2012;13:77–80.

[19] Pochini AC , Rodrigues MSB, Yamashita L, Belangero PS, Andreoli CV, Ejnisman B. Surgical treatment of pectoralis major muscle rupture with adjustable cortical button. Rev Bras Ortop 2018;53:60–6. 29367908 10.1016/j.rboe.2017.11.005 PMC5771794

[20] Pochini AC , Andreoli CV, Ejnisman B, Maffulli N. Surgical repair of a rupture of the pectoralis major muscle. BMJ Case Rep 2015;2015:pii:bcr201320229. 10.1136/bcr-2013-202292 PMC4342657 25716033

[21] Pochini AC , Ferretti M, Kawakami EF. Analysis of pectoralis major tendon in weightlifting athletes using ultrasonography and elastography. Einstein (Sao Paulo) 2015;13:541–6. 26761551 10.1590/S1679-45082015AO3335 PMC4878628

[22] Rabuck SJ , Lynch JL, Guo X. Biomechanical comparison of 3 methods to repair pectoralis major ruptures. Am J Sports Med 2012;40:1635–40. 22679296 10.1177/0363546512449291

[23] Schachter AK , White BJ, Namkoong S, Sherman O. Revision reconstruction of a pectoralis major tendon rupture using hamstring autograft: a case report. Am J Sports Med 2006;34:295–8. 16170043 10.1177/0363546505278697

[24] Goutallier D , Postel JM, Bernageau J, Lavau L, Voisin MC. Fatty muscle degeneration in cuff ruptures. Pre- and postoperative evaluation by CT scan. Clin Orthop Relat Res 1994;304:78–83. 8020238

[25] Schepsis AA , Grafe MW, Jones HP, Lemos MJ. Rupture of the pectoralis major muscle. Outcome after repair of acute and chronic injuries. Am J Sports Med 2000;28:9–15. 10653537 10.1177/03635465000280012701

[26] Heikkinen J , Lantto I, Piilonen J. Tendon length, calf muscle atrophy, and strength deficit after acute achilles tendon rupture: long-term follow-up of patients in a previous study. J Bone Joint Surg Am 2017;99:1509–15. 28926379 10.2106/JBJS.16.01491

[27] Hozack MJW , Bugg B, Lemay K, Reed J. Tears of pectoralis major in steer wrestlers: a novel repair technique using the EndoButton. Clin J Sport Med 2013;23:80–2. 23103785 10.1097/JSM.0b013e318272ca52

[28] Tietjen R . Closed injuries of the pectoralis major muscle. J Trauma 1980;20:262–4. 7359604 10.1097/00005373-198003000-00015

[29] Gruber AJ , Pope HG Jr. Compulsive weight lifting and anabolic drug abuse among women rape victims. Compr Psychiatry 1999;40:273–7. 10.1016/s0010-440x(99)90127-x. 10428186

[30] Sephien A , Orr J, Remaley DT. Pectoralis major tear in a 23-year-old woman while performing high-intensity interval training: a rare presentation. BMJ Case Rep 2020;18:13. 10.1136/bcr-2019-232649 PMC7101035 32193177

[31] Ejnisman B , Filho J, Andreoll CVI, Monteiro GC, Pochini AC, Cohen M. Piomiosite multifocal em atleta: relato de caso. Rev Bras Ortop 2007;42:157–60.

[32] Macedo C , Dornelles L, et al. Uso de esteroides anabolizantes em praticantes de musculação e/ou fisiculturismo. Rev Bras Med Esporte 1998;4:13–7.

